# Why and how should we promote home dialysis for patients with end-stage kidney disease during and after the coronavirus 2019 disease pandemic? A French perspective

**DOI:** 10.1007/s40620-021-01061-7

**Published:** 2021-06-01

**Authors:** Guy Rostoker, Belkacem Issad, Hafedh Fessi, Ziad A. Massy

**Affiliations:** 1 Department of Nephrology and Dialysis, Hôpital Privé Claude Galien, Ramsay Santé, Quincy-sous-Sénart, France; 2 Collège de Médecine des Hôpitaux de Paris, Paris, France; 3 Peritoneal Dialysis Centre, Nephrology Department, Groupe Hospitalier Pitié-Salpêtrière, AP-HP, Paris, France; 4 Home Haemodialysis Unit, Nephrology and Dialysis Department, Hôpital Tenon AP-HP, Paris, France; 5 Nephrology Department, Hôpital Ambroise Paré, APHP, Boulogne; and INSERM Unit 1018, CESP, University Versailles-St Quentin, University Paris Saclay, Villejuif, France

**Keywords:** Home Dialysis, Peritoneal Dialysis, Home Haemodialysis, COVID-19, SARS-CoV-2

## Abstract

The health crisis induced by the pandemic of coronavirus 2019 disease (COVID-19) has had a major impact on dialysis patients in France. The incidence of infection with acute respiratory syndrome coronavirus 2 (SARS-CoV-2) during the first wave of the COVID-19 epidemic was 3.3% among dialysis patients—13 times higher than in the general population. The corresponding mortality rate was high, reaching 21%. As of 19th April, 2021, the cumulative prevalence of SARS-CoV-2 infection in French dialysis patients was 14%. Convergent scientific data from France, Italy, the United Kingdom and Canada show that home dialysis reduces the risk of SARS-CoV-2 infection by a factor of at least two. Unfortunately, home dialysis in France is not sufficiently developed: the proportion of dialysis patients being treated at home is only 7%. The obstacles to the provision of home care for patients with end-stage kidney disease in France include (i) an unfavourable pricing policy for home haemodialysis and nurse visits for assisted peritoneal dialysis (PD), (ii) insufficient training in home dialysis for nephrologists, (iii) the small number of administrative authorizations for home dialysis programs, and (iv) a lack of structured, objective information on renal replacement therapies for patients with advanced chronic kidney disease (CKD). We propose a number of pragmatic initiatives that could be simultaneously enacted to improve the situation in three areas: (i) the provision of objective information on renal replacement therapies for patients with advanced CKD, (ii) wider authorization of home dialysis networks and (iii) price increases in favour of home dialysis procedures.

## Introduction: reasons why home dialysis should now be a priority in France

Healthcare systems in France, in Europe, and around the world have been hit hard by the ongoing pandemic of coronavirus 2019 disease (COVID-19), caused by severe acute respiratory syndrome coronavirus 2 (SARS-CoV-2). Worldwide, dialysis patients have paid a heavy price during the COVID-19 pandemic, and the crisis has also had a major impact on the nurses and physicians providing these patients with medical care. Although this new viral disease has revealed the strengths, unprecedented flexibility, and resilience of the French and European healthcare systems, it has also highlighted limitations in their organizational structures and funding systems.

The COVID-19 crisis prompted us as nephrologists to discuss the “post-COVID-19 world”.

We sought to develop pragmatic recommendations that would complement the overall guidelines on home dialysis issued by the French-speaking Society of Nephrology, Dialysis and Transplantation (SFNDT) [1]—which we fully endorse—and a report by the French Court of Audit [2], both of which were drawn up before the COVID-19 pandemic. Given the many similarities among healthcare systems in the European Union (EU)’s 27 member states, we believe that these thoughts and proposals will interest nephrologists throughout Europe.

## What is the current state of treatment for patients with end-stage kidney disease in France?

Chronic kidney disease (CKD) is a major public health problem in France: of the nearly 3 million individuals affected by this condition, around 90,000 have end-stage kidney disease (ESKD), 40,421 have received a kidney transplant, and 49,271 patients require dialysis [3, 4].

At present, 93% of these dialysis patients attend in-hospital or satellite dialysis centres [3, 4].

Home dialysis is an alternative to in-centre haemodialysis and currently covers two techniques: peritoneal dialysis (PD) and home haemodialysis (HHD). For non-autonomous patients and the very elderly, assisted PD is performed by a nurse who travels to the patient’s home. Assisted PD has been reimbursed by the French national social security system for more than 30 years.

HHD is performed by autonomous patients five to seven days of the week for 120 to 150 min per session. The growth of this technique over the last 10 years has been stimulated by the availability of specific miniaturized dialysis machines.

Home dialysis has been widely developed in a number of European countries; it concerns 25% of dialysis patients with ESKD in Sweden, 21% in the Netherlands, and 19% in the United Kingdom [1–3]. In contrast, home dialysis is barely progressing in France, where it currently concerns only 7% of dialysis patients (6% on PD and 1% on HHD) [1, 2, 4]. Similarly, low figures for home dialysis are observed in Germany (6%) and Portugal (7%), whereas other European countries have recently succeeded in increasing the proportion of patients on home dialysis: 9% in Austria, 10% in both Belgium and in Italy [1, 2, 4].

## Dialysis patients have paid a heavy price during the COVID-19 pandemic

Compared with the general population, dialysis patients in France have been particularly exposed to the risk of contracting COVID-19. This is essentially because of their compulsory thrice-weekly attendance at dialysis centres, the obligatorily long dialysis session (4–5 h), the rarity of single-patient dialysis rooms (most dialysis centres in France and other EU countries have communal rooms), and the use of ambulances to travel to and from the centre. In France, the incidence of infection with SARS-CoV-2 during the first wave of the COVID-19 epidemic was 3.3% among dialysis patients—13 times higher than in the general population [5].

Furthermore, dialysis patients are typically elderly (the current median age in France is 71 years [4]) and tend to have a high co-morbidity burden (particular diabetes mellitus, arterial hypertension and cardiovascular disease [4]). Along with the immune deficiency induced by ESKD, these factors led nephrologists to fear that dialysis patients could present a severe form of COVID-19 – as seen with other infectious diseases. The initial data indicate that these fears were well founded: an analysis of the REIN register by the French Biomedicine Agency (*Agence de la Biomédecine*) for the period between March 16th and May 4th, 2020, highlighted 1621 cases of COVID-19 and 344 deaths among the country’s 48,669 dialysis patients [5]. This corresponds to a very high infection rate (nearly 3.3%, vs. 0.2% in the general population in France) and a high mortality rate in symptomatic cases (21%, vs. 14.7% in the general population; cumulative number of patients infected in France in May 2020 the 2sd: 167,346 with 24,594 deaths) [5]. These results were confirmed by an analysis of the European Renal Association-European Dialysis and Transplant Association (ERA-EDTA) COVID-19 Database (ERACODA) for 26 countries (mainly in Europe) and 98 dialysis centres: between February 1st and May 1st, 2020, 1073 cases of symptomatic, PCR-positive COVID-19 were collated in patients with ESKD (305 kidney transplant patients and 768 dialysis patients) [6]. In Europe, the probability of dying within 28 days of a diagnosis of COVID-19 appears to be very high among kidney transplant patients (21.3%) and even higher among dialysis patients (25%) [6].

Of note, as of the 19th April, 2021, 7,096 French dialysis patients have contracted COVID-19; thus, the cumulative prevalence of SARS-CoV-2 infection is estimated by the REIN register in France to be 14% among dialysis patients [7].

In contrast, recent data from the French home dialysis register (RDPLF) [8] and from the Register of Piedmont and Aosta Valley in Italy [9] clearly show that dialysis patients treated at home (PD, in this case) were only half or a third as likely to contract COVID-19 during the first wave. In France, only 59 (1.8%) of the 3,104 patients on PD at 156 units contracted the disease [8]. In Italy, only 4 (1%) of the 387 patients on PD in Piedmont and Aosta Valley contracted a SARS-CoV-2 infection vs. 3.4% (98 out of 2,893) of the in-centre haemodialysis patients [9]. A protective effect of PD (vs. centre-based haemodialysis) has also been demonstrated in Canada and England, with an approximately three-fold reduction in the risk of infection [10]. In the latter country (where the COVID-19 epidemic was particularly intense), the infection rate during the first wave was 9% among haemodialysis patients treated in centres and just 2.9% among PD patients [10].

A recent editorial in the *Journal of Nephrology* combined data from the Biomedicine Agency’s REIN register and the RDPLF register on HHD in France during the first wave. The proportion of HHD patients with symptomatic COVID-19 (1.65%, i.e., 7 out of 423) was similar to that observed for PD patients [11]. This relative reduction in the risk of infection was even greater in the Ile-de-France region, with an infection rate of 3.6% (4 out of 109) among HHD patients and 11.5% (930 out of 8,025) among patients undergoing centre-based haemodialysis (*p* = 0.001) [11].

It is noteworthy that on April 22nd, 2020, the Italian Ministry of Health wisely published a guideline asking healthcare professionals to implement measures to increase the use of HHD and PD through appropriate patient education programs, in order to minimize the risk of contracting COVID-19 among dialysis patients [12]. Similar recommendations have also been compellingly advocated in the *Journal of the American Society of Nephrology* [10] and the *Journal of the Italian Society of Nephrology* [13]. A prevention strategy for this population makes sense, since the results of mathematical modelling by a group of Harvard epidemiologists (published in the journal *Science*) suggested that COVID-19 will persist for several more years–even after mass vaccination – and will continue to produce significant seasonal peaks until 2024 [14].

In view of these convergent scientific data, we recommend the development of home dialysis as a blood purification method that appears to protect patients against infection by SARS-CoV-2.

## Home dialysis as the optimal method for patients in outer suburbs, again favoured as residential areas

Residential areas in the outer suburbs (i.e., far from city centres) are once again very popular among younger generations in France and elsewhere in Europe – especially following the periods of lockdown and the introduction of teleworking in many sectors that accompanied the waves of COVID-19 [15]. It would not make sense to set up new satellite dialysis centres in these outer suburbs. For future patients with ESKD living in these remote areas, home dialysis (HHD and automated PD in particular) appears to be the most rational response in view of the individuals’ educational level, use of teleworking, adequate housing, and desire for autonomy.

## Planning and providing dialysis for very elderly patients

The pandemic has revealed an unexpectedly high degree of intergenerational solidarity while highlighting profound flaws in the organizational structure and funding of institutions for dependent elderly people in the various member countries of the Organization for Economic Co-operation and Development (OECD) [16].

Moreover, nephrologists in France and elsewhere in Europe must also anticipate the massive arrival of the baby-boomer generation (i.e., people born between 1945 and 1955) as early as 2025–just as the COVID-19 pandemic will probably start to recede [16]. Home dialysis teams in France and elsewhere in Europe have now acquired significant, positive experience of how home PD (usually continuous ambulatory peritoneal dialysis (CAPD)) can empower couples of active octogenarians living in their own home, and how assisted PD can benefit nonagenarians who alternate between living in their own home and living with their children. Furthermore, feedback from home dialysis teams in Toronto, Canada, shows that it is possible to implement HHD for elderly patients by paying younger family members or care assistants [17]. Thus, a worldwide demographic explosion of very elderly patients with ESKD will follow the COVID-19 pandemic. The need to maintain a normal, reassuring living environment for these elderly patients can only be met by home dialysis (i.e., assisted PD and assisted haemodialysis).

## Why is home dialysis underexploited in France?

The obstacles to the development of home care for patients with ESKD in France have been known for years now [1–3]:

- For HHD, an unfavourable pricing policy has made hospital and dialysis network managers reluctant to promote a low-margin technique. This is combined with a lack of homogeneity in fees for nephrologists between dialysis techniques, with HHD being the only blood purification procedure in France without any medical fees (in the non-profit and for-profit sectors). This two-fold financial handicap is accentuated by the outdated requirement for the presence of another person during the dialysis session. Lastly, there are regulatory and financial restrictions on home visits by a nurse to patients who are reluctant to puncture (or not very skilled in puncturing) their fistula.
- For assisted PD, one of the main obstacles is currently the low-level reimbursement of a home visit by a nurse for peritoneal exchanges and catheter care.
- Unfortunately, there are many other obstacles to the development of home dialysis in France. Firstly, medical training on PD and HDD is often still limited and varies markedly from one region to another. Secondly, administrative authorizations of home dialysis (both PD and HHD) for health establishments (whether public or private) are not fluently given by France’s regional health agencies (*agences régionales de santé* (ARS)) – except in the Ile-de-France region. Lastly, and prior to the very recent (October 2019) introduction of a national CKD care pathway, information on the various renal replacement therapies for patients with advanced CKD was not structured in an objective, homogeneous way in France.

## Several easily applicable initiatives should enable the rapid development of home dialysis in France

The above analysis of obstacles to the development of home dialysis in France shows that it is necessary to work pragmatically and simultaneously to improve matters in three areas: (i) the provision of objective information on renal replacement therapy to patients with advanced CKD, (ii) the implementation of home dialysis techniques in a greater number of areas, and (iii) an increase in the pricing of these techniques.

A CKD care pathway was set up by the French Ministry of Health for public-sector, non-profit and for-profit health establishments in October 2019. The pathway’s main goals are to provide objective information to patients with advanced CKD (stages 4 and 5) on the various renal replacement techniques (including transplantation and home dialysis), and thus to enable patients to make a truly informed choice in this respect. It is noteworthy that the French Health Authority (*Haute Autorité de Santé* (HAS)) has published detailed information sheets on dialysis and transplantation to be given to patients with advanced CKD (stages 4 and 5), together with specific diaries for patient follow-up at stages 4 and 5 by nephrologists, specialist nurses, and dieticians [18].

Unfortunately, the pathway’s initial implementation was heavily impaired by the COVID-19 epidemic.

The pathway generates not only process indicators but also outcome indicators, such as the proportion of advanced CKD patients stabilized without dialysis and the proportion who opt for home dialysis or pre-emptive familial transplantation.

Once fully deployed, the CKD care pathway will probably break the non-virtuous, self-sustaining cycle of centre-based dialysis that has prevailed until now. Important issues for the CKD care pathway include the influence of health literacy on the choice of home dialysis and thus the use of specifically designed media and educational tools to overcome this known cause of suboptimal patient care in CKD [19, 20].

We also believe that the revival of home dialysis techniques in France should be driven by a more democratic, broader process for authorizing home dialysis programs for applicant health establishments from all sectors (public, non-profit, and for-profit), with support from the ARSs.

Furthermore, pricing increases appear to be necessary for the development of home dialysis techniques, as highlighted by the SFNDT’s white paper [1]; this involves the currently undervalued packages for HHD sessions, the creation of a medical package for weekly HHD monitoring (just like the one that has existed for several years for PD), the creation of a home nursing procedure for fistula puncture and connection to the dialysis machine, and the abolition of the requirement for the presence of a partner during an HHD session. Likewise, price increases for PD-related home nursing procedures for non-autonomous patients is a prerequisite for the major extension of a technique that will enable us to manage the coming “generational crisis” among elderly patients with CKD.

Home dialysis techniques significantly decrease the carbon footprint—mainly by reducing the use of transportation, relative to in-centre or satellite haemodialysis. Moreover, daily home haemodialysis with low-flow dialysate (3 h a day, 5.5 days a week) has been shown to generate only 1.8 tons of CO_2_ equivalent per patient per year, vs. 3.8 tons of CO_2_ equivalent per patient per year for conventional, thrice-weekly, 4.5-h in-centre haemodialysis [21, 22]. However, the carbon footprints of HHD and PD could be further reduced with a view to meeting “green dialysis” standards by (i) decreasing the volume of consumables and dialysate and (ii) using more environmentally friendly packaging [21, 23].

## Conclusion

We believe that some of our thoughts and proposals will be of interest to nephrologists working in European countries where, as in France, home dialysis is insufficiently deployed.
